# Anomalous Scaling of Gene Expression in Confined Cell-Free Reactions

**DOI:** 10.1038/s41598-018-25532-3

**Published:** 2018-05-09

**Authors:** Ryota Sakamoto, Vincent Noireaux, Yusuke T. Maeda

**Affiliations:** 1 Department of Physics, Kyushu University, Motooka 744, Fukuoka, 819-0395 Japan; 2 School of Physics and Astronomy, University of Minnesota, 115 Union street, Minneapolis, MN 55455 USA

## Abstract

Cellular surface breaks the symmetry of molecular diffusion across membrane. Here, we study how steric interactions between the surface and the bulk of cell-sized emulsion droplets alters gene expression emulated by a cell-free transcription/translation (TXTL) system. The concentration of synthesized reporter proteins in droplets of radius R shows an anomalous geometric scaling of *R*^4^ different from the expected size-dependence of *R*^3^. Given that TXTL becomes less efficient at thin surface layer, a mathematical model explains the anomalous size-dependence found in experiment. The surface of cell-sized compartment can thus play a regulatory role for cell-free gene expression.

## Introduction

As expressed by W. Pauli “God made the bulk, surfaces were invented by the devil.”, the surface has remarkable properties due to its anisotropic nature, especially in microscopic systems. The atoms or molecules close to the surface have three different types of interaction: with the inner phase, with the outer phase and with the neighbors on the surface. The fact that broken symmetry in surface alters the material properties has been widely accepted in condensed matter physics, from semiconductors^1^ to colloidal transport^2^. In biological systems, the smallest functional and structural unit, which has a functional bulk space enclosed by a thin interface, is a living cell. The typical size of living cells ranges from 1 to 100 *μ*m^3^. Consequently, the surface area to volume ratio (S/V ratio) plays an important role for cellular physiology. On the one hand, lasting exposure to the environment enhances the nutrient or water uptake with higher efficiency^4^.

On the other hand, the surface of cells faces the inner cytosolic space in which genetic information stored in DNA can be expressed through transcription (TX) and translation (TL)^5,6^ Cell-free transcription-translation (TXTL), such as the PURE system^7,8^ or TXTL^9–13^ recapitulates gene expression *in vitro*. TXTL have become ideal platforms to construct cellular functions in isolation through the expression of synthetic gene circuits. TXTL, used in this work, is based on a cytoplasmic extract prepared from *E. coli* that contains the molecular machineries for efficient transcription and translation. TXTL is increasingly used to engineer models of synthetic cells^14–17^ Cell-free reactions of both PURE system and TXTL are also encapsulated into cell-sized emulsion droplets or liposomes programmed with gene circuits for a specific cellular function^18–21^ At the fundamental level, however, a lot remains to be understood on how gene expression is altered in such microscopic compartments. While the variability in the kinetics of cell-free gene expression in confined environments has been extensively discussed^22–30^ the functional interplay between the surface, TXTL reactions, and intracellular metabolism remains elusive and poorly characterized.

In this study, we demonstrate that cell-sized compartments alter the geometric scaling law of gene expression in TXTL. We derive a model to show that the geometric scaling between produced protein and the radius of a compartment *R* reflects surface-induced gene regulation: when surface represses or activates gene expression, the protein amount scales with *R*^4^ or *R*^2^ respectively. Experimentally, we show that the concentration of a synthesized reporter protein increases in a quartic manner as a function of droplet radius when TXTL is executed in cell-sized emulsion droplets. This anomalous geometric scaling supports interfacial repression of TXTL. Our findings provide new insight for minimal bioreactor in a cell-sized space.

## Results

### Cell-free gene expression in droplets

**Figure 1:**
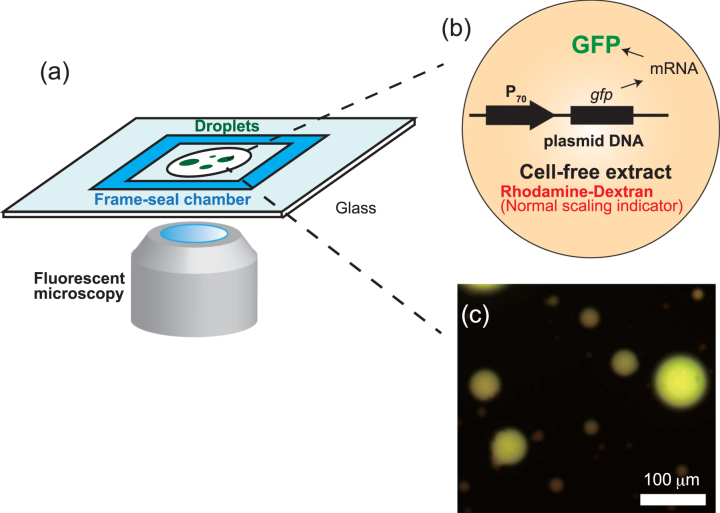
(**a**) Schematic illustration of experimental setup. Emulsion droplets containing the TXTL reaction and plasmid DNA are recorded in multi-color fluorescent microscopy. (**b** and **c**) The product of TXTL, deGFP, was observed in green fluorescent channel while Rhodamine-dextran as normal scaling indicator was visualized in red-fluorescent channel. Scale bar: 100 *μ*m.

The TXTL system used is this work (Fig. 1) was described previously^11,12^ TXTL contains all the necessary proteins and metabolites to achieve efficient CFGE (cell-free gene expression). The plasmid P_70*a*_-deGFP (3.2 kbp, used at 16 nM, final conc. 3.5 nM)^11^ was used to express the reporter protein deGFP (green fluorescent protein). Rhodamine-dextran (Sigma-Aldrich) was also used as a red fluorescent marker to verify the normal geometric scaling (∝*R*^3^). The emulsion droplets were prepared using the phospholipid 18:1 (Δ9-Cis) phosphatidyl-choline (DOPC, Avanti Polar Lipids) as surfactant in mineral oil (Sigma-Aldrich). The DOPC lipid was dissolved in the fluorinated oil at 0.1% (w/v) so as to make reaction-in-oil droplet (DOPC droplet) coated by a lipid monolayer. DOPC droplets encapsulating TXTL reaction with DNA were prepared by adding 30 *μ*L lipid-dissolved oil to 12 *μ*L TXTL reaction, then emulsified by tapping on the tube. The 10 *μ*L of the emulsion was placed onto a microscope coverglass with a polydimethyl-siloxane coating and enclosed by a frame seal chamber with a cover slip. Time-lapse of GFP fluorescence kinetics were recorded at time interval of 5 min for 15 hours by epi-fluorescence (IX73, Olympus microscope) with a cooled CMOS camera (Neo5.5, Andor). The size of droplets was analyzed by a custom-made MATLAB image processing. The average number of plasmid DNA is around 1000 within a droplet of its radius *R* = 5 *μ*m (0.5 pL in volume). To avoid large statistical fluctuations of DNA copy number among DOPC droplets of *R* ≥ 5 *μ*m were subject to image analysis. The temperature was kept at 31 °C using a custom-made hot water bath attached to the microscope stage.

**Figure 2:**
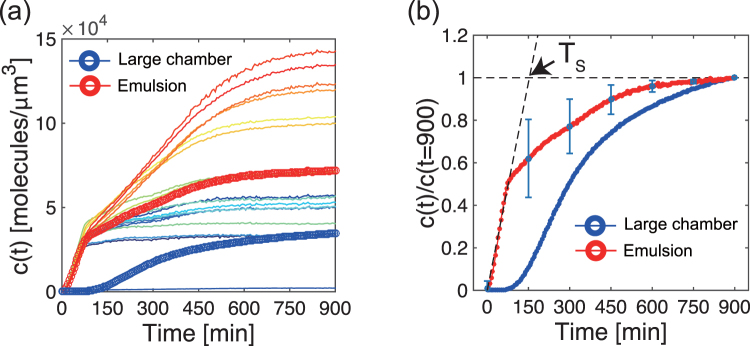
(**a**) Time-course of protein synthesis (GFP concentration, *c*(*t*)) in various size of 18 droplets. The averaged kinetics over 18 droplets is plotted in red circle. Kinetics of deGFP production in TXTL that was performed in a large frame-seal chamber is also shown in blue circle (reaction volume was around 10  *μ*L). (**b**) The time-courses of confined microscopic TXTL reactions averaged over DOPC droplets (standard deviation is shown as blue vertical bars) and large volume TXTL reactions (performed in chamber) are normalized by the end-point GFP intensity. This curve was used to estimate the saturation time, *T*_*s*_, in mathematical model.

Poly-dispersed droplets, with diameter ranging from 10 to 100 *μ*m, were prepared to characterize CFGE of a reporter gene in confined environment.Expression of the reporter gene *degfp* was driven by the promoter P_70_ (Fig. 1(b))^11^. The overall system recapitulates gene expression *in vitro* in physiological conditions so as to mimick conditions found in living cells^14^. Figure 1(c) shows DOPC droplets containing the synthesized deGFP after 15 h of incubation with Rhodamine-dextran. To determine the dynamics of CFGE in the emulsion droplets, we measured the time-course of deGFP accumulation. As shown in Fig. 2, the time-course of deGFP fluorescence in emulsion exhibited a monotonic increase, each curve corresponds to single droplets varied in size. The fluorescence level reached plateau after 15 hours, indicating that all the TXTL reactions have stopped.

### Anomalous scaling of cell-free gene expression

**Figure 3:**
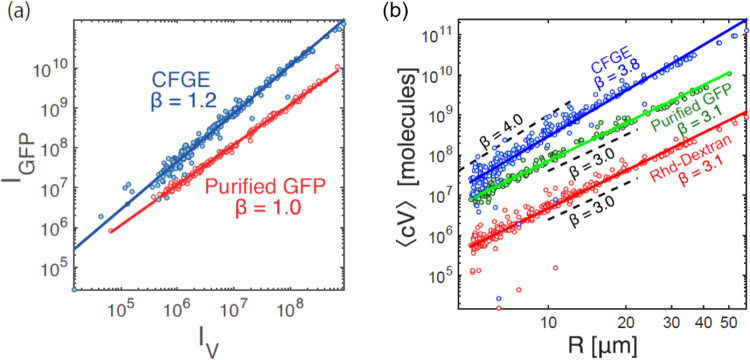
(**a**) deGFP expressed from TXTL extract (N = 246) and purified deGFP (control, N = 191) in each droplet. The horizontal axis represents the droplet size by using fluorescent marker (Rhodamine-Dextran). (**b**) Log-log plots of the total intensity along droplet radius. Blue circles denote deGFP intensity resulting from CFGE in emulsion droplets. Green circles and red circles are the intensity of purified deGFP and marker protein, respectively.

The total fluorescence intensity *I*_*v*_ from Rhodamine-dextran (concentration *c*_*Rhd*_) scaled proportionally to the volume of each droplets *V*, so that normal scaling was observed as *I*_*v*_ = *c*_*Rhd*_*V* ∝ *R*^3^ (Fig. 3(a)). Interestingly, the signal *I*_*GFP*_, which is the total fluorescence intensity of deGFP in each droplet, exhibited non-linear dependence to the normal scaling indicator *I*_*v*_ as shown in Fig. 3(a). The *I*_*GFP*_ for the expressed deGFP from TXTL is ${I}_{GFP}\propto {I}_{v}^{\beta }$ with *β* = 1.2, meaning that it is not linearly scaled with respect to the volume though *I*_*GFP*_ for purified deGFP is proportionally increased with the normal scaling indicator *I*_*v*_. This nonlinear dependence indicates that CFGE is less efficient in smaller droplets. This result revealed an unexpected geometric dependence of CFGE when TXTL is executed in emulsion droplet.

To characterize this size-dependent CFGE, we further examined *I*_*GFP*_ in size-dependent manner. We encapsulated in the emulsion droplets a mixture of the TXTL reaction, plasmid DNA and Rhodamine-dextran in order to perform dual-color fluorescent imaging of synthesized deGFP by CFGE and Rhodamine-dextran in the same experiment. Figure 3(b) shows *I*_*GFP*_ of expressed deGFP as a function of the radius of each droplet. We obtained a scaling function of *I*_*GFP*_ ∝ *R*^*β*^ with *β* = 3.8, suggesting that deGFP synthesized inside droplets follows an anomalous geometric scaling different from the ordinal *R*^3^ dependence. In contrast to expressed deGFP, the total fluorescence intensity of Rhodamine-dextran in emulsion droplets exhibited *I*_*v*_ ∝ *R*^3.1^, proportional to the volume (Fig. 3(b), red circle). Our next control experiments consisted of encapsulating purified deGFP protein inside the emulsion droplets. The *I*_*GFP*_ of purified deGFP still had *R*^3^ dependence, identical to the Rhodamine-dextran *I*_*v*_ ∝ *R*^3.1^. This control experiment indicated that the anomalous scaling *I*_*GFP*_ ∝ *R*^3.8^ is uniquely observed when deGFP is synthesized through TXTL inside the emulsion droplets.

### Mathematical model

CFGE from the TXTL reactions has been described by Karzbrun, *et al*.^14^. Their coarse-grained mathematical model proposes a first order kinetics for mRNA degradation while the protein degradation follows a zeroth-order kinetics when deGFP is tagged for proteolysis. With no tag, deGFP cannot be degraded (infinite lifetime)^14^ (see Supplementary Fig. S1). We extended this model for spherical confinement in order to describe the mechanism underlying in size-dependent CFGE.

**Figure 4:**
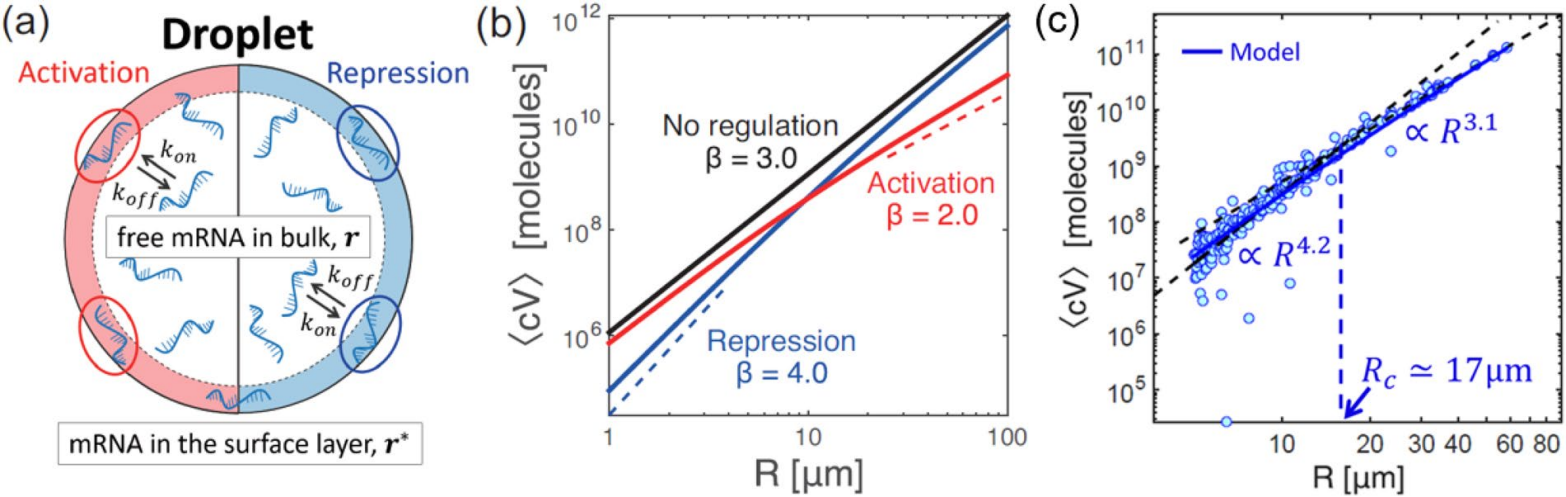
(**a**) Schematic illustration of the surface regulation model in a cell-sized compartment. (**b**) Theoretical analysis of anomalous scaling of produced protein 〈*cV*〉 in confined cell-free systems. Three types of regulation regime show different size-dependent scaling. Repression (blue, $\alpha \gg {\alpha }^{\ast }$), no regulation (black, *k*_*on*_ = 0), and activation (red, $\alpha \ll {\alpha }^{\ast }$) correspond to *β* = 4.0, *β* = 3.0, and *β* = 2.0 for 〈*cV*〉 ∝ *R*^*β*^ respectively (broken lines are guide for the eyes). (**c**) Total produced protein number 〈*cV*〉 is plotted along radius of droplet *R*. The data shown are the same as CFGE in Fig. 3(b) (blue circles), and Eq. (6) is fitted to them with a least-square regression (blue solid curve). Black broken lines are fitted to the data points *R* < *R*_*c*_ and *R* > *R*_*c*_, and the corresponding slopes are shown as the texts *R*^4.2^ and *R*^3.1^ respectively.

Consider a cell-sized compartment of radius *R*, in which CFGE takes place and the products of transcription (TX) and translation (TL) cannot diffuse across the surface. Hereafter, we assume that protein synthesis in the vicinity of the droplet surface (thickness *λ* ≈ 30 nm^31^) is regulated due to the activation/inactivation of TXTL onto the surface (Fig. 4(a)). We first give the mathematical model of gene expression under confinement. The kinetics of free mRNA (*r*), regulated mRNA (r^*^) and synthesized protein (*c*) are described by the following rate equations:

1 $$\frac{dr}{dt}={k}_{R}-{k}_{on}r\frac{3\lambda }{R}+{k}_{off}{r}^{\ast }-{\gamma }_{R}r,$$

2 $$\frac{d{r}^{\ast }}{dt}={k}_{on}r\frac{3\lambda }{R}-{k}_{off}{r}^{\ast }-{\gamma }_{R}^{\ast }{r}^{\ast },$$

3 $$\frac{dc}{dt}=\alpha r+{\alpha }^{\ast }{r}^{\ast }.$$

where the transcription rate of mRNA from DNA is *k*_*R*_, and the degradation rate of free mRNA and regulated mRNA are denoted as *γ*_*R*_ and ${\gamma }_{R}^{\ast }$ respectively. mRNA produced from transcription is adsorbed to the thin surface layer of water-oil interface with the attachment rate *k*_*on*_ and the dissociation rate *k*_*off*_. The S/V ratio of $4\pi {R}^{2}\lambda /\frac{4}{3}\pi {R}^{3}\to \frac{3\lambda }{R}$ determines the capacity for mRNA in the surface layer, so that it leads the factor of 3*λ*/*R* in Eqs (1) and (2).

Synthesis of mRNAs rapidly reaches steady-state values 〈*r*〉 and 〈r^*^〉 due to the short lifetime of mRNA molecules with respect to the observation time^14^. Steady-state expression is obtained by setting *dr*/*dt* = 0, dr^*^/*dt* = 0:

4 $$\langle r\rangle =\frac{{r}_{0}}{\frac{3\lambda }{R}\frac{\theta }{\tau }+1},\,\langle {r}^{\ast }\rangle =\frac{3\lambda }{\tau R}\cdot \frac{{r}_{0}}{\frac{3\lambda }{R}\frac{\theta }{\tau }+1},$$

where *r*_0_ = *k*_*R*_/*γ*_*R*_, $\theta ={\gamma }_{R}^{\ast }/{\gamma }_{R}$, $\tau =({k}_{off}+{\gamma }_{R}^{\ast })/{k}_{on}$. Multiply $V=\frac{4}{3}\pi {R}^{3}$ to Eq. (3) and substitute Eq. (4) into Eq. (3), then integrate it with the initial condition of *c*(0) = 0 and we obtain the time dependence of protein concentration:

5 $$c(t)V=V(\alpha \langle r\rangle +{\alpha }^{\ast }\langle {r}^{\ast }\rangle )t=\frac{{r}_{0}V}{\frac{3\lambda }{R}\frac{\theta }{\tau }+1}(\alpha +{\alpha }^{\ast }\frac{3\lambda }{\tau R})t.$$

In our experimental setup, protein concentration finally saturates at a certain value due to the depletion of nutrients^14^. Hence we determined the saturation time *T*_*s*_ = 153 [min] from the intersection point between the extrapolation of Eq. (5) and the experimentally observed plateau value (Fig. 2(b)). Therefore, in the steady-state Eq. (5) is rewritten as

6 $$\langle cV\rangle =\frac{{r}_{0}V}{\frac{3\lambda }{R}\frac{\theta }{\tau }+1}(\alpha +{\alpha }^{\ast }\frac{3\lambda }{\tau R}){T}_{s}.$$

Equation (6) represents the general geometric scaling of CFGE under confinement, so that we next examine the exponents of size-dependent scaling under two kinds of gene regulation, namely either surface-induced repression ($\alpha \gg {\alpha }^{\ast }$) or surface-induced activation ($\alpha \ll {\alpha }^{\ast }$). First, for the surface-induced repression of TXTL, given that $\alpha \gg {\alpha }^{\ast }$ yields as the repressed protein synthesis, Eq. (6) becomes

7 $$\langle cV\rangle \simeq \frac{4}{3}\pi {r}_{0}\alpha {T}_{s}\cdot \frac{{R}^{3}}{1+\frac{3\lambda }{R}\frac{\theta }{\tau }}.$$

To find a simple scaling relation, one expects that the following relation holds: $\theta \gg \tau$, i.e. ${\gamma }_{R}\approx {\gamma }_{R}^{\ast }$ and ${k}_{on}\gg {k}_{off}+{\gamma }_{R}^{\ast }$, meaning that the translation of mRNA is suppressed at the surface until mRNA is degraded. This approximation gives a quartic dependence of CFGE on *R*: 〈*cV*〉 ∝ *R*^4^ which tells translation inhibition for small droplets is significant, while the proportion of surface-induced repression becomes negligible for large droplet and approaches *R*^3^. We found that this size-dependent scaling Eq. (7) agrees well with the experimental result shown in Fig. 4(c), suggesting *I*_*GFP*_ ∝ *R*^4^ for CFGE in DOPC droplets. We thus conclude that TXTL reactions in emulsion droplets belongs to the class of surface-induced repression.

Next, we examined whether translation could be enhanced at the surface of the droplets. If mRNA translation at the surface layer is enhanced compared to mRNA in the bulk ($\alpha \ll {\alpha }^{\ast }$), Eq. (6) becomes

8 $$\langle cV\rangle \simeq \frac{\frac{4}{3}\pi {r}_{0}{\alpha }^{\ast }{T}_{s}}{\theta }\cdot \frac{{R}^{3}}{1+\frac{R}{3\lambda }\frac{\tau }{\theta }}.$$

For large droplets, Eq. (8) becomes 〈*cV*〉 ∝ *R*^2^, whereas size-dependence becomes *R*^3^ for small droplets. This kind of size-dependence was not observed in our experiment. These two anomalous scalings of 〈*cV*〉 are shown in Fig. 4(b). A simple mathematical model thus proposes how the surface induces a size-dependent scaling of protein synthesis.

As for surface-repression regime, the key concept in the mathematical model is a thin depletion layer at the interface water-oil of the droplets. Ribosomes, which are the major component responsible for translation, are depleted from the membrane surface due to steric repulsion. The translation of deGFP within the depletion layer is assumed to become less efficient as $\alpha \gg {\alpha }^{\ast }$. Because the depth of the depletion layer *λ* is comparable to the size of a ribosome, i.e. *λ* = 30 nm^31^, this parameter is incorporated in the mathematical model. By taking into account the depletion-mediated translation repression, the experimental data was fitted to the proposed mathematical model with a least-square regression (Fig. 4(c) and Table 1).

**Table 1:** List of parameters used for model fitting (blue solid curve) in Fig. 4(c).

Symbol	Name	Value
*k*_*R*_	Transcription rate of mRNA	3.22 × 10^−3^ [*μ*m^−3^ s^−1^]
*k*_*on*_	Attachment rate of free mRNA	1.00 [s^−1^]
*k*_*off*_	Dissociation rate of regulated mRNA	3.86 × 10^−3^ [s^−1^]
*γ*_*R*_	Degradation rate of free mRNA	1.39 × 10^−3^ [s^−1^]^14^
${\gamma }_{R}^{\ast }$	Degradation rate of regulated mRNA	1.39 × 10^−3^ [s^−1^]^14^
*α*	Translation rate of free mRNA	9.02 [s^−1^]
*α* ^*^	Translation rate of regulated mRNA	1.96 × 10^−1^ [s^−1^]
*T*_*s*_	Saturation time	153 [min]
*λ*	Thickness of surface regulation layer	30 [nm]^31^

The biochemical parameters used for the model were chosen or determined as follows. In addition to the depth of surface region *λ*, there are four given parameters, *k*_*on*_, *γ*_*R*_ and ${\gamma }_{R}^{\ast }$, and *T*_*s*_. Attachment rate was set as *k*_*on*_ = 1.0 [s^−1^] to consider the condition where the fraction of *r*⋅(3*λ*/*R*) mRNA is regulated in the depletion layer (Eq. (1)). Degradation rate *γ*_*R*_ was already known in the literature^14^, in which the same TXTL system from *E. coli* was studied and ${\gamma }_{R}^{\ast }$ was assumed to be the same as *γ*_*R*_. The saturation time *T*_*s*_ was obtained from Fig. 1(b). Using these parameters, we determined the rest of free parameters, *k*_*R*_, *k*_*off*_, *α* and α^*^ with a least-square regression. The obtained *k*_*R*_ was consistent with the known value^14^. The translation rates of *α* and α^*^ are one order of magnitude larger than the values in the literature^14^. We hypothesize that it is because emulsified droplets at *R* = 5 to 50 μm were surrounded by fluorinated oil in which large amounts of oxygen are dissolved, enabling more efficient supply of oxygen required for translation^9^. Moreover, the condition ${k}_{on}\gg {k}_{off}$ also supported the anomalous scaling of *R*^4^ as well as ${k}_{on}\gg {\gamma }_{R}^{\ast }$.

Furthermore, we analyzed the crossover of geometric scaling from *R*^4^ to *R*^3^ predicted in the mathematical model. We defined the characteristic radius *R*_*c*_ by $\frac{3\lambda \theta }{{R}_{c}\tau }=1$ over which 〈*cV*〉 enters normal scaling of *R*^3^ in Eq. (7). By using relevant parameters, *R*_*c*_ is rewritten as

9 $${R}_{c}=3\lambda \frac{{\gamma }_{R}^{\ast }}{{\gamma }_{R}}\cdot \frac{{k}_{on}}{{k}_{off}+{\gamma }_{R}^{\ast }}.$$

As shown in Fig. 4(c), using the parameter set, the crossover radius in this experiment is *R*_*c*_ = 17 *μ*m. One finds the anomalous scaling of *R*^4.2^ at *R* < *R*_*c*_ while the normal scaling of *R*^3.1^ at *R* > *R*_*c*_. The theoretically predicted anomalous scaling and crossover were observed in our experiments. We therefore conclude that our theoretical description reasonably captures the main feature of the surface repression regime of confined CFGE.

## Discussion

It is well established that electrostatic interactions can make polyelectrolytes, such as DNA, adsorb to charged surfaces^32^. Our finding show that geometric regulation of CFGE occurs even at an interface rich in neutral lipids. We hypothesize that steric interaction alone at thin surface layer (typically thickness of a few tens of nm) can give rise to control CFGE. Although such surface-induced repression of TXTL in a cell-sized droplet is observed in emulsified droplet, CFGE from the PURE system in liposomes^33,34^ appears to show normal scaling behavior of *I*_*GFP*_ ∝ *R*^3^. The PURE system has greater stability against mRNA degradation rather extract-based system^35,36^ the approximation ${k}_{on}\gg {k}_{off}+{\gamma }_{R}^{\ast }$ is reasonable as well. Such discrepancy may result from the difference of physical nature of CFGE expression systems: TXTL, used in this work, is based on an extract that is rather crowded due to the presence of macromolecules (10 mg/ml of proteins and 2% PEG8000). On the other hand, liposomes containing the PURE system in which macromolecules are relatively diluted will be difficult to support the surface layer, implying that the rate of translation close to the membrane surface could be comparable to that in bulk (*α* ≈ α^*^). The experimental verification of surface-induced activation, *I*_*GFP*_ ∝ *R*^2^, will be addressed in future work.

The anomalous scaling of gene expression points out that confinement with stable surface can alter the protein synthesis even in simple cell-free systems. This fact in turn brings practical strategy to design how the functional surface of artificial bioreactors capable of rational control of gene expression. For small compartment as small as bacteria, because the S/V ratio is large, the regulation of CFGE associated with membrane becomes more effective. For surface-repression strategy, confinement in small droplets may be useful to save chemical resources for protein synthesis. When the droplets grow or coalesce until their size becomes sufficiently large^19,37^ the droplets will recover normal size-dependence of *R*^3^. In contrast, to suppress the protein synthesis at larger droplets, surface-induced activation is favorable due to another anomalous scaling of *R*^2^. Thus, the sign of surface-induced regulation can be important to build functional interface in confined cell-free bioreactors.

As for further implication, the characteristic size *R*_*c*_ = 17 *μ*m at which the crossover of geometric scaling from *R*^4^ to *R*^3^ occurs may be relevant to CFGE reactions executed in growing droplets. As long as a droplet has a radius smaller than *R*_*c*_, surface-induced translation inhibition decreases protein synthesis, which saves energy resources as discussed above. Once the droplet grows larger than *R*_*c*_, either by coalescence or by autonomous growth, it enters the normal scaling regime for translation. Beyond such example, our results could be also relevant to other applications such as TXTL *in vitro* evolution experiments performed in similar emulsion droplets^38,39^.

## Conclusion

In this study, we demonstrated that cell-free TXTL of a reporter gene executed in cell-sized spherical emulsion droplets of 10–100 *μ*m diameter shows anomalous geometric scaling of *R*^4^, which differs from the expected scaling behavior of *R*^3^. To explain the observed scaling behavior, we propose a mathematical model for confined cell-free gene expression where steric repulsion of the translational machinery from membrane surface is considered. Such steric effect within a thin surface layer makes translation reaction less efficient. The membrane surface negatively regulates TXTL at large surface to volume ratio. A crossover between a negatively regulated TXTL and normal TXTL is observed at *R*_*c*_ = 17 *μ*m. Our finding raises the importance of physical size in micro-scale compartments where cell-free systems are encapsulated. Further investigation of CFGE in microscopic compartments will provide better understanding of confinement-induced enzymatic kinetics in a cell-sized space^10,18^ and in turn will advance our knowledge about miniaturized factories engineered for parallel synthesis of proteins in large populations of droplets^9,15,29,30^.

## Methods

### Image analysis

Quantitative image analysis was done using custom code written in MATLAB (see Supplementary Fig. S2). Specifically, the bright field image taken at end point was processed through binarization and in turn the center of mass of each droplets was detected. To obtain the radius of droplets of *R*, the area of each droplets were extracted and then assumed to *πR*^2^. To determine *R* with better precision and reliability, we performed two filtering processes. When the water-in-oil droplets spread onto the substrate surface due to wetting or when the coalescence of multiple droplets occurred, such droplets exhibited deformed ellipsoid-like shape. Because such asymmetry causes potential difficulties in subsequent data analysis, we excluded the debris according to two filtering methods. The first filtering method was used to figure out whether detected objects were spherical droplets or deformed one. We defined a metric, *M*, to evaluate the roundness of an object: *M* = 4*π* × *S*/*L*^2^, where *S* and *L* is the area and the perimeter of the object, respectively. This metric becomes 1 for a spherical object while less than 1 for any other shape. Its precision was adjusted with an appropriate threshold and we employed threshold *M* = 0.75 in this study. The second filtering method consisted of removing small debris of micelles out of the detected objects. The detected objects whose size was less than 78.6 *μ*m^2^ were excluded from subsequent analysis. This filtering process sets the minimum limit in droplet radius at 5 *μ*m. In addition, background intensity was subtracted from each pictures, where background region was chosen in a way to be sure no background pixels were in droplets.

### Protein purification

Purified deGFP with a 6-histidine-tag at the C-terminal end was used in one of the control experiments. The deGFP gene was cloned into the pET28b(+) vector using conventional cloning methods. deGFP was over-expressed in the *Escherichia coli* strain BL21(DE3)pLysS (TaKaRa) and then purified through Ni-NTA column (QIAGEN). After measuring the concentration of purified deGFP by both Bradford assay and gel-electrophoresis, the calibration curve of fluorescent intensity of deGFP in fluorescent microscopy and protein concentration was obtained. The concentration of deGFP was determined using the molar extinction coefficient of 55000 M^−1^ cm^−1^.

## Electronic supplementary material

Supplementary information
