# Supplementary material for: Anomalous Scaling of Gene Expression in Confined Cell-Free Reactions
Source: Sci Rep. 2018 May 9;8:7364. doi: 10.1038/s41598-018-25532-3 (PMC5943292; doi:10.1038/s41598-018-25532-3)
Supplement: Supplementary file 1 — Supplementary information [file 41598_2018_25532_MOESM1_ESM.pdf]

Supplemental information for
Anomalous Scaling of Gene Expression in Confined Cell-Free Reactions

Ryota Sakamoto[1,*], Vincent Noireaux[2], and Yusuke T. Maeda[1]

[1]Kyushu University, Department of Physics, Motooka 744, Fukuoka 819-0395, Japan.
[2]University of Minnesota, School of Physics and Astronomy 115 Union street, Minneapolis, MN 55455 USA
*sakaryo@phys.kyushu-u.ac.jp

Experimental details

Protein degradation in confined cell free extract

It has been shown that protein degradation in cell free extract is zeroth order[1]. As for GFP reporter protein used in this study, protein degradation is assumed to be negligible owing to the absence of ssrA peptide tag responsible for protein degradation. To test whether the degradation of GFP is negligibly small, we measured the decay of fluorescent intensity of purified deGFP in the TXTL extract within DOPC droplets. The time course of fluorescent intensity is shown in Figure S1(a), where data curve is normalized by the end-point (900 min) thereafter averaged over all droplets (red curve) and standard deviation is shown (blue vertical bars). Intensity fluctuation is observed in the time course due to the slight moving of each droplet, however, there is no considerable intensity decrement. In addition, we also tested the decay of purified GFP protein in the phosphate buffer solution within DOPC droplets but its considerable reduction was negligible (Figure S1(b)). We thus conclude that there is no active degradation in TXTL extract as well in this study.

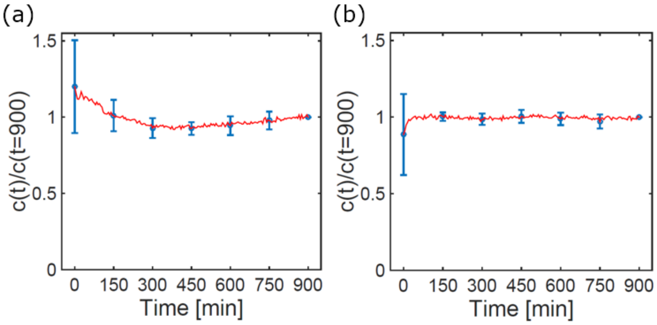

Figure S1. (a) Time course of normalized GFP intensity in water-in-oil droplets comprising of TXTL extract and purified deGFP, (b) comprising of PBS (Phosphate-buffered saline) and purified deGFP. The red curve is average for over all droplets ((a): $N = 29$, (b): $N = 24$) and standard deviation is shown as blue vertical bars.

Image analysis

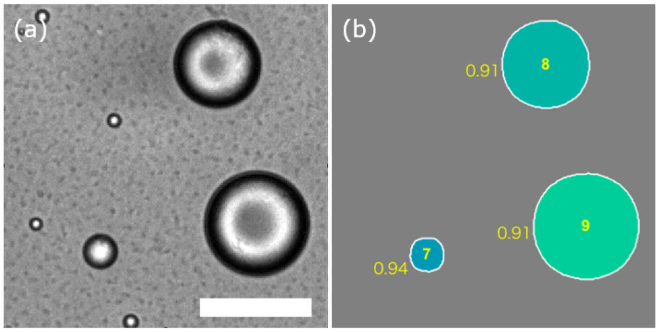

Figure S2. (a) GFP fluorescence within droplets (bar: 40 μm). (b) Droplets are labeled by the numbers in the center of each droplet. Three-digit numbers outside of the droplets denote the metric of each droplet. The small debris and coalesced droplets were excluded after filtering methods in Figure S2(b).

References

1. Karzburn, E., Shin, J., Bar-Ziv, R.H., & Noireaux, V. Coarse-Grained Dynamics of Protein Synthesis in a Cell-Free System. *Phys. Rev. Lett.* **106**, 048104 (2011).
